# Supplementary material for: The Koala (Phascolarctos cinereus) faecal microbiome differs with diet in a wild population
Source: PeerJ. 2019 Apr 1;7:e6534. doi: 10.7717/peerj.6534 (PMC6448554; doi:10.7717/peerj.6534)
Supplement: Supplemental Information 1 [file peerj-07-6534-s001.docx]

**The Koala (*Phascolarctos cinereus*) faecal microbiome differs with diet in a wild koala population.**

Kylie L. Brice^1,3^, Pankaj Trivedi^1,2,3^, Thomas C. Jeffries^1,2^, Michaela D. J. Blyton^1^, Christopher Mitchell^1,4^, Brajesh K. Singh^1,2^, Ben D. Moore^1^*.

^1^Hawkesbury Institute for the Environment and ^2^Global Centre for Land Based Innovation Western Sydney University, Locked bag 1797 Penrith, NSW, 2751, Australia. ^3^Colorado State University, Bioagricultural Sciences & Pest Management, C023 Plant Sciences Building, Fort Collins, 80523-1177, Colorado, United States of America. ^4^Environment & Sustainability Institute, University of Exeter, Penryn, Cornwall, United Kingdom, TR10 9FE.

Corresponding authors: Kylie Brice, Email: kylie.brice@colostate.edu, Phone +1 9704023556. Ben Moore, Email: b.moore@westernsydney.edu.au, Phone +61 (0)2 4570 1384.

**Supplementary Information**

**Methods**

**Faecal collection**

Mats were placed under koalas observed residing in trees within areas dominated by either *E. viminalis* or *E. obliqua* (see Fig. S1 and S2). The Global Positioning System (GPS) locations were recorded once a koala was observed residing in a tree, and tree identification was determined (and recorded), using a hand-held Garmin eTrex 10 GPS unit (Garmin Australia, Eastern Creek, NSW). Waypoints were created for each koala samples were collected from following manufacturer’s instructions.

*Determination of microbial community composition and relative abundance*

*DNA extraction*

Microbial DNA was extracted from three faecal pellets (technical replicates) per koala using the MoBio PowerSoil® DNA isolation kit (Mobio Laboratories, Carlsbad, California, USA) as per the manufacturer’s instructions and as described by (Costello *et al.,* 2009). Briefly, 0.25 grams (g) of faeces (obtained from inside the pellet), was added to the 750 microliters (µl) bead bed, 100 µl of sterilized milli-Q ultra-pure water and 160 µl of the C1 solution were added and tubes were vortexed on high for 10 minutes (min). Final elution consisted of 100 µl of sterile nuclease-free water (Invitrogen, Mulgrave, VIC, Australia). DNA quality was quantified by Qubit 2.0 Fluorometric assay (Life Technologies, Carlsbad, California, USA).

*Terminal restriction fragment length polymorphism (T-RFLP)*

All PCR products obtained from individual technical replicates for bacterial and archaeal 16S rRNA gene DNA and fungal ITS DNA were purified separately using the Wizard® SV Gel and PCR clean-up system (Promega, Sydney, NSW, Australia) following the DNA purification by centrifugation protocol. After purification, PCR product concentrations were determined by NanoDrop (Thermo Fisher Scientific). All sample concentrations were standardised to 300 ng / µl. Purified replicates underwent digestion separately using the Hha1 restriction enzyme (Genesearch Pty. Ltd, Arundel, QLD, Australia); each reaction contained 300 ng purified PCR products, 1xNEBuffer, 10 U of Hha1 restriction enzyme (Genesearch) and 2 µg BSA (Genesearch). Appropriate negative controls were used; reaction volumes were adjusted to a total of 10 µl with milli-Q ultra-pure water (Merck Millipore). Samples were incubated using the Dyad Disciple™ Pelter Thermal Cycler (Bio-Rad) at 37° C (3 h) and deactivated at 95° C (10 min). Terminal restriction fragment length polymorphism analyses was undertaken, targeting bacteria, archaea and fungus using primers and conditions described by Singh et al. (2006a). one µl of each DNA extract was submitted for T-RFLP analysis using the ABI 3500 Genetic Analyser (Applied Bio Systems®/Life Technologies Australia) at the Hawkesbury Institute for the Environment (Western Sydney University, Australia).

**Diet analysis**

*n-alkane analysis*

Briefly, 0.2 ± 0.01 g of plant or faecal material was weighed out in duplicate into glass scintillation vials and extracted overnight in 1.5 ml of ethanolic potassium hydroxide solution (1 M) containing 0.11 ± 0.01 g *n*-docosane (C_22_) and *n*-tetratriacontane (C_34_) in *n*-decane as the internal standard. Alkane analysis was carried out using a 30 m HP-5ms column (Agilent Technologies Pty Ltd, Mulgrave, VIC, Australia) with an internal diameter of 0.25 mm and a film thickness of 0.25 μm (Agilent Technologies), operated with hydrogen as a carrier gas at a flow rate of 1.2 ml min −1. Two microliters of extract were injected into a multimode inlet operating in split mode at 280°C, with a split ratio of 10:1 and an initial oven temperature of 170 °C. The initial temperature was held for 4 min before increasing at a rate of 30 °C min−1 to 215°C and then at 6°C min−1 to 300°C holding for 3 min. The transfer line operated at 280°C, the MS scan range was set from 40 m/z to 600 m/z.

**Data analysis**

*Bacterial and fungal rRNA community fingerprinting by T-RFLP analysis*

Raw T-RFLP data were analysed with GeneMapper v5 (Applied Bio Systems®/Life Technologies). Peak presence/absence data were generated and exported for further statistical analysis as described by Singh *et al.,* (2006b). All multivariate statistical analyses of T-RFLP data were conducted using PRIMER v 7.0.13 (Clarke, 1993) and permutational multivariate analysis of variance (PERMANOVA+, Anderson, 2001 PRIMER-E, Plymouth, UK). Data underwent standardisation (by sample total-default setting) and square root transformation; taxonomic distinctness was assessed using the Bray-Curtis dissimilarity measure (Bray and Curtis, 1957). To test for differences in microbial community composition between koalas eating *E. viminalis* and koalas eating *E. obliqua*, we used PERMANOVA+ (Anderson, 2001), PERMANOVA models used for analysis of collection, consisted of fixed (collection) × random (diet), for analysis of individual collection year, fixed (diet) × random (koala) respectively. Principal co-ordinates analysis (PCoA) plots were used to visualise differences in microbial community composition and structure within and between the koalas using R studio v 3.3.1 (R Development Core Team, 2013) using the ggplot2 package (Wickham, 2009).

*Parabacteroides* and Ruminococcaceae *phylogenetic tree construction*

The *Parabacteroides* and Ruminococcaceae phylogenetic trees were constructed using 16S rRNA gene sequences available through NCBIs BLASTn database (previously identified genomes), as well as the 16S rRNA sequences identified during our study. Sequences were initially aligned utilising CLUSTALW (Thompson *et al.,* 1994), the phylogenetic trees were constructed utilising the Phylogeny.fr platform in the ‘one click’ mode (Dereeper *et al.,* 2008), which proceeds through a predefined pipeline including MUSCLE multiple alignment (Edgar 2004). Trees were constructed using phylogenetic maximum-likelihood (PhyML; Guindon & Gascuel 2003) which uses the approximate Likelihood Ratio statistical test for branches (aLRT v3.0), thereby enabling reliability of internal branch length to be assessed (Anisimova & Gascuel 2006; Dereeper *et al.,* 2008). Trees were graphically rendered by TreeDyn (v198.3; Chevenet *et al.,* 2006; Dereeper *et al.,* 2008) within the Phylogeny fr. pipeline and exported in Newick format and visualised using the Mega6 (v6) software (Tamura *et al.,* 2013).

*Diet composition and quality analysis*

Foliage was collected from *E. viminalis* and *E. obliqua* to profile cuticular n-alkane markers for comparison with faecal marker profiles, allowing us to confirm each individual’s diet composition. These foliage samples were also assessed for nutritional quality.

**Results**

***Bacterial and fungal rRNA community fingerprinting***

**Analysis of bacterial T-RFLP 16S rRNA gene using the beta diversity metric,** Bray-Curtis **and principal coordinates analysis (PCoA), indicated a clearly defined separation occurring between koalas eating *E. viminalis* and those eating *E. obliqua,* with 22% of the total variation explained by the PC1, there was also a separation occurring along the principal component (PC) 2 axis (17%, Fig. S3), which accounted for the total variation between the combined collection years (2013 and 2015). Assessment of the** Bray-Curtis metric **using PERMANOVA indicated no influence of collection year (*Pseudo* *F_2_*=1.19 PERMANOVA P=0.49 Fig S3), although it detected a significant influence of diet (*Pseudo* *F_2_*=11.36, PERMANOVA P=0.0001 Fig S3).**

**PCoA plots of the individual collection years, based on the** Bray-Curtis **matrices, also demonstrate significant separation occurring between the koalas eating *E. viminalis* and those eating *E. obliqua* in 2013, with 45% of the total variation explained by the first PC axis (*Pseudo F_1_*=11.75, PERMANOVA P=0.0001; Fig*.* S4**a)**. The bacterial 16S rRNA gene TRFs for the 2015 collection showed a lesser, but significant, separation (*Pseudo* *F_1_*=5.13, PERMANOVA P=0.0001, Fig. S4**b), with the first two principal components explaining 38% of total variation.  **PERMANOVA analysis of the Archaeal 16S rRNA T-RFLP data for the 2013 koala collection detected an influence of diet (*Pseudo* *F_1_*=4.82, PERMANOVA P=0.0001; Fig. S6a). Analysis of fungal samples from the 2013 koala collection demonstrated a significant influence of diet (*Pseudo* *F_1_*=2.41, PERMANOVA P=0.004; Fig. S6b). PCoA scatterplots for archaea identified a small separation between the two diets for archaea, with PC1 and PC2 explaining 38% of variation (*Fig.* S6a). No separation was detected in the PCoA analysis for fungus (*Fig.* S6b). Further investigation revealed a separation on PC3 (9.3%). As the most clearly defined separation occurred within the bacterial community, we proceeded to further sequence only the 16S rRNA gene in this study.**

*Functional differences between previously identified Parabacteroides and Ruminococcaceae genomes*

The analysis of 35 previously identified *Parabacteroides* and 45 Ruminococcaceae genomes identified GH families involved in the degradation of plant cell walls, starch and other components ranging from easily degraded to recalcitrant (Fig. S7). In general, *Parabacteroides* genomes (associated with *E. viminalis* diets) possess more genes for oligosaccharide degradation than Ruminococcaceae genomes, while Ruminococcaceae genomes (more strongly associated with *E. obliqua* diets) possess up to five times the number of enzymatic genes targeting the degradation of recalcitrant cellulose (Fig. S7). *Parabacteroides* genomes also have more genes from the GH67 and GH85 families, which are involved in the degradation of xylan and chitin, respectively. The genomic potential to degrade tannins appears higher in *Parabacteroides*, while ATP-binding cassette (ABC) and phosphotransferase system (PTS) transporter genes were more common in Ruminococcaceae (average of 134 ABC and 18 PTS per genome, Table S5) than in *Parabacteroides* (82 ABC and 6 PTS, Table S5).

**References**

Anderson, M.J. (2001) A new method for non‐parametric multivariate analysis of variance. *Austral Ecol.* **26**: 32-46.

Anisimova, M., and Gascuel, O. (2006) Approximate likelihood ratio test for branches: a fast, accurate and powerful alternative. *Syst. Biol.* **55**: 539-552.

Berlemont, R., and Martiny, A.C. (2015) Genomic potential for polysaccharide deconstruction in bacteria. *Appl. Environ. Microbiol.* **81**: 1513-1519.

Bray, J.R., and Curtis, J.T. (1957) An ordination of the upland forest communities of southern Wisconsin. *Ecol. Monograph.* **27**: 325-349.

Cantarel, B.L., Coutinho, P.M., Rancurel, C., Bernard, T., Lombard, V., and Henrissat, B. (2009) The Carbohydrate-Active EnZymes database (CAZy): an expert resource for glycogenomics. *Nucleic Acids Res.* **37**: D233-D238.

Chevenet, F., Brun, C., Bañuls, A.L., Jacq, B., Christen, R. (2006) TreeDyn: towards dynamic graphics and annotations for analyses of trees. *BMC bioinformatics*. **7**:439.

Clarke, K.R. (1993) Non‐parametric multivariate analyses of changes in community structure. *Aust. J. Ecol.* **18**: 117-143.

Costello, E.K., Lauber, C.L., Hamady, M., Fierer, N., Gordon, J.I., and Knight, R. (2009) Bacterial community variation in human body habitats across space and time. *Sci.* **326**: 1694-1697.

Dereeper, A., Guignon, V., Blanc, G., Audic, S., Buffet, S., Chevenet, F., Dufayard, J. F., Guindon, S., Lefort, V., Lescot, M., Claverie, J. M., Gascuel, O. (2008) Phylogeny. fr: robust phylogenetic analysis for the non-specialist. *Nucleic Acids Res.* **3**: W465–W469.

Edgar, R.C. (2004) MUSCLE: multiple sequence alignment with high accuracy and high throughput. *Nucleic acids res*. **32**:1792-1797.

Guindon, S. and Gascuel, O. (2003) A simple, fast, and accurate algorithm to estimate large phylogenies by maximum likelihood. *Syst. Biol.* **52**: 696-704.

R Development Core Team (2013) R: A language and environment for statistical computing. In *R Foundation for Statistical Computing (2013)*.

Singh, B.K., Nazaries, L., Munro, S., Anderson, I.C., and Campbell, C.D. (2006b) Use of multiplex terminal restriction fragment length polymorphism for rapid and simultaneous analysis of different components of the soil microbial community. *Appl Environ Microbiol* **72**: 7278-7285.

Tamura, K., Stecher, G., Peterson, D., Filipski, A. and Kumar, S., 2013. MEGA6: molecular evolutionary genetics analysis version 6.0. *Mol. Biol. Evol.* **30**: 2725-2729.

Thompson, J. D., Higgins, D. G., Gibson, T. J. (1994) CLUSTALW: Improving the sensitivity of progressive multiple sequence alignment through sequence weighting, position-specific gap penalties and weight matrix choice. *Nucleic Acids Res.* **22**: 4673-4680.

Wickham, H. (2009) *ggplot2: elegant graphics for data analysis*: Springer Science & Business Media.
