# Supplementary figures and images for: The Koala (Phascolarctos cinereus) faecal microbiome differs with diet in a wild population
Source: PeerJ. 2019 Apr 1;7:e6534. doi: 10.7717/peerj.6534 (PMC6448554; doi:10.7717/peerj.6534)

Legend

- *Eucalyptus viminalis*
- *Eucalyptus obliqua*

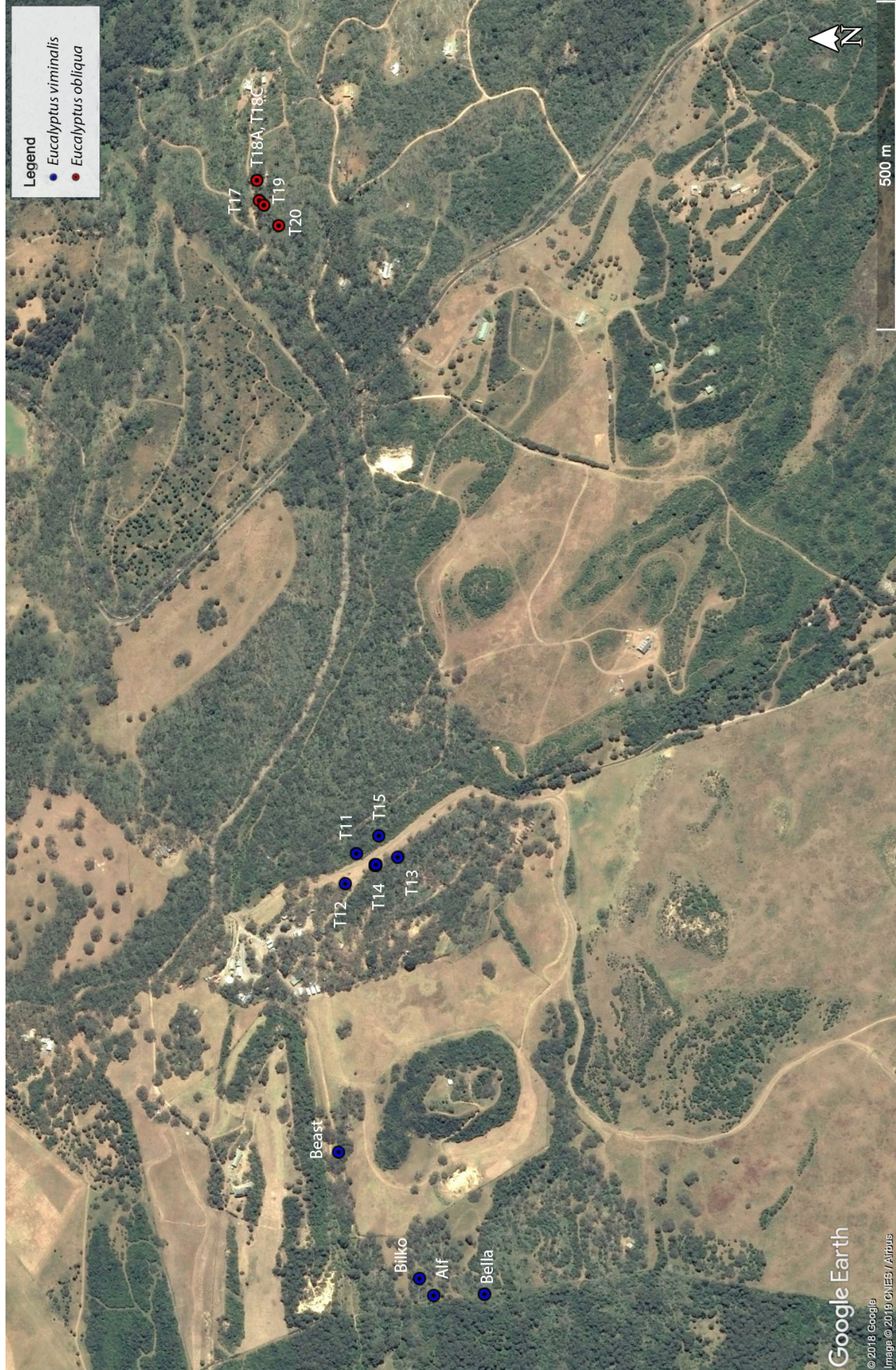

Supplement: Figure S1 — Map showing locations of the Cape Otway 2013 koalas located in E. viminalis and E. obliqua trees, within discreet areas of E. viminalis and E. obliqua forest patches. GPS locations were taken at collection during 2013, Google Earth map produced 10th November 2018 (Map data ©2018 Google). [file peerj-07-6534-s002.pdf]

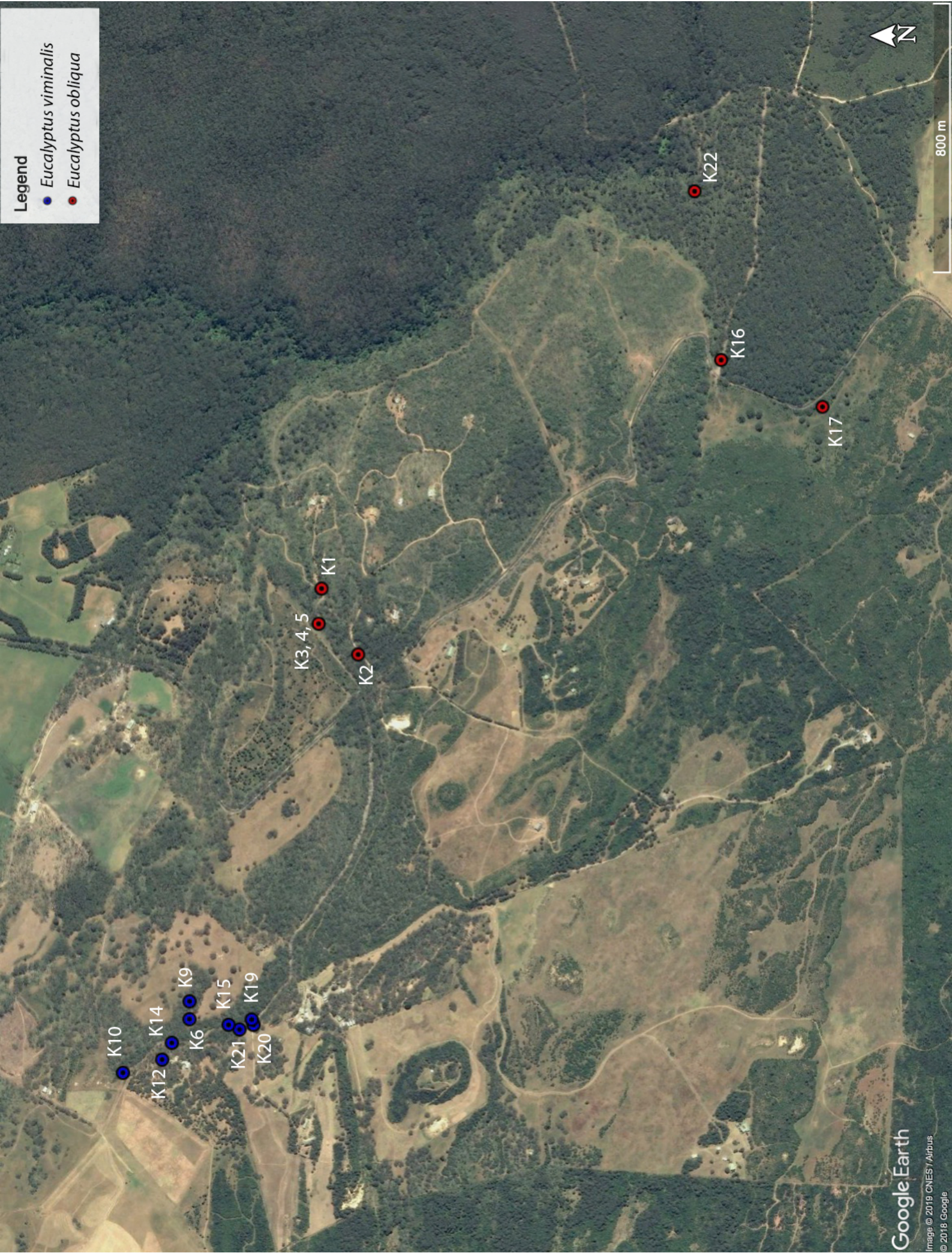

Legend

● *Eucalyptus viminalis*

● *Eucalyptus obliqua*

Supplement: Figure S2 — Map showing locations of the Cape Otway 2015 koalas located in E. viminalis and E. obliqua trees, within discreet areas of E. viminalis and E. obliqua forest patches. GPS locations were taken at collection during 2015. The GPS coordinates for the koala identified as K13 from the 2015 collection were not recorded, and therefore it is not shown on the map. Its faeces were sampled in the same area as the other E. viminalis koalas. Google Earth map produced 10th November 2018 (Map data ©2018 Google). [file peerj-07-6534-s003.pdf]

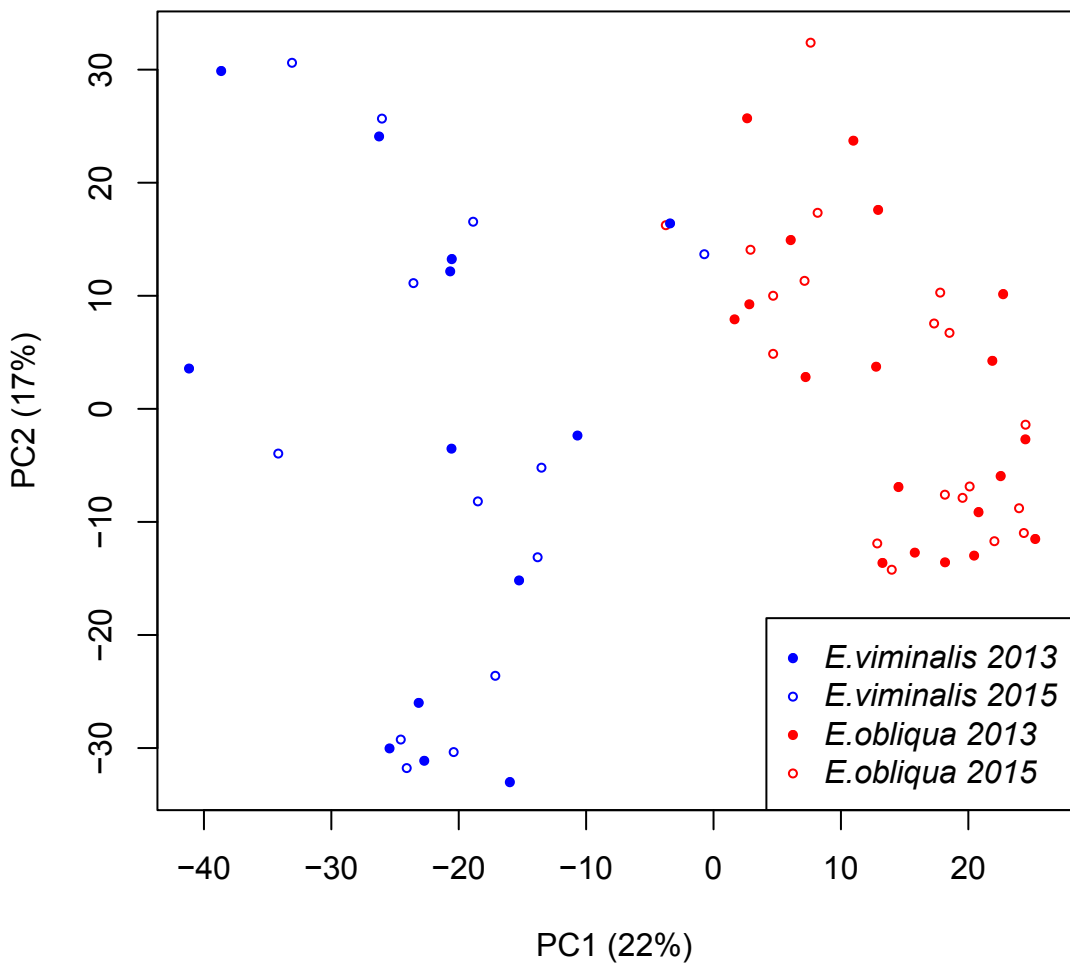

Supplement: Figure S4 — Scatterplot from the Bray–Curtis metric for the combined Cape Otway koala population with diets comprising E. viminalis and E. obliqua. Assessment of the Bray–Curtis metric using PERMANOVA indicated no influence of collection year (Pseudo F2 = 1.19 PERMANOVA P = 0.49), although it detected a significant influence of diet (Pseudo F2 = 11.36, PERMANOVA P = 0.0001). [file peerj-07-6534-s004.pdf]

2013

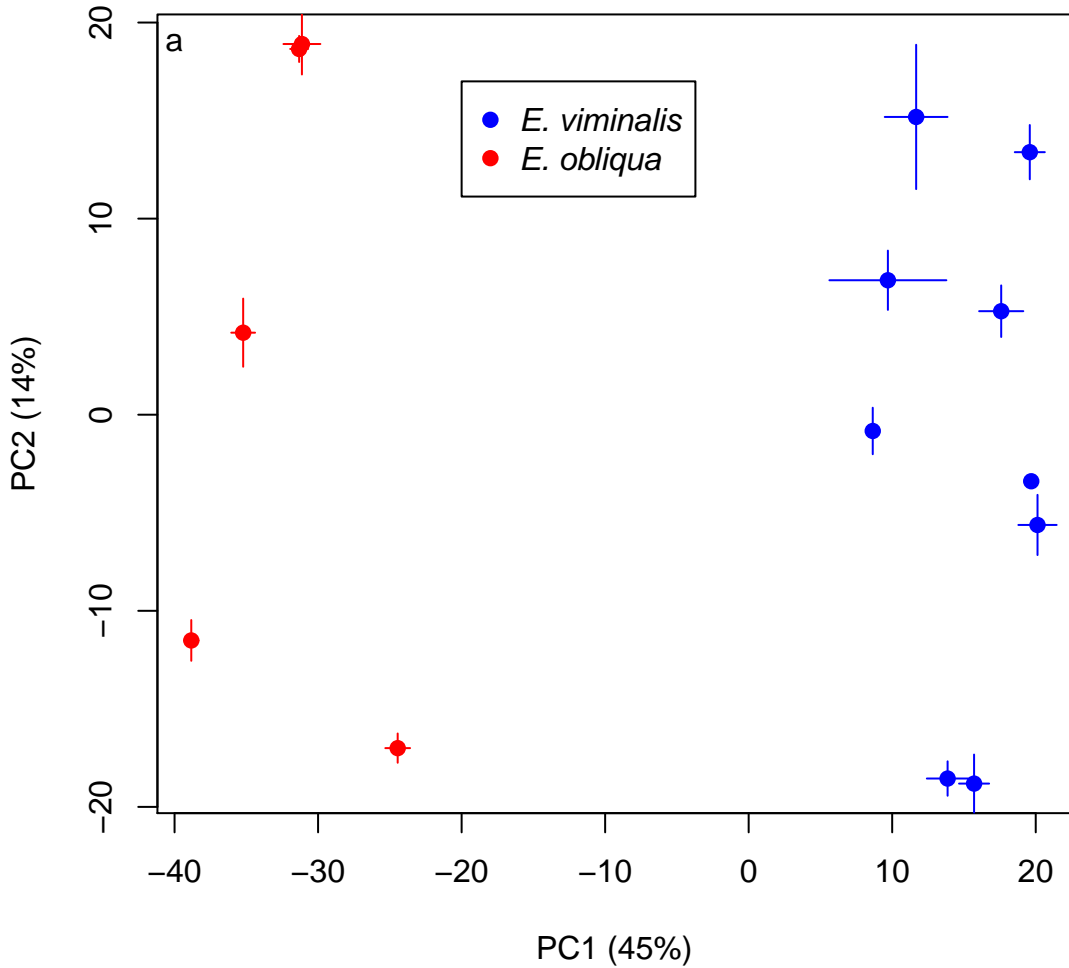

2015

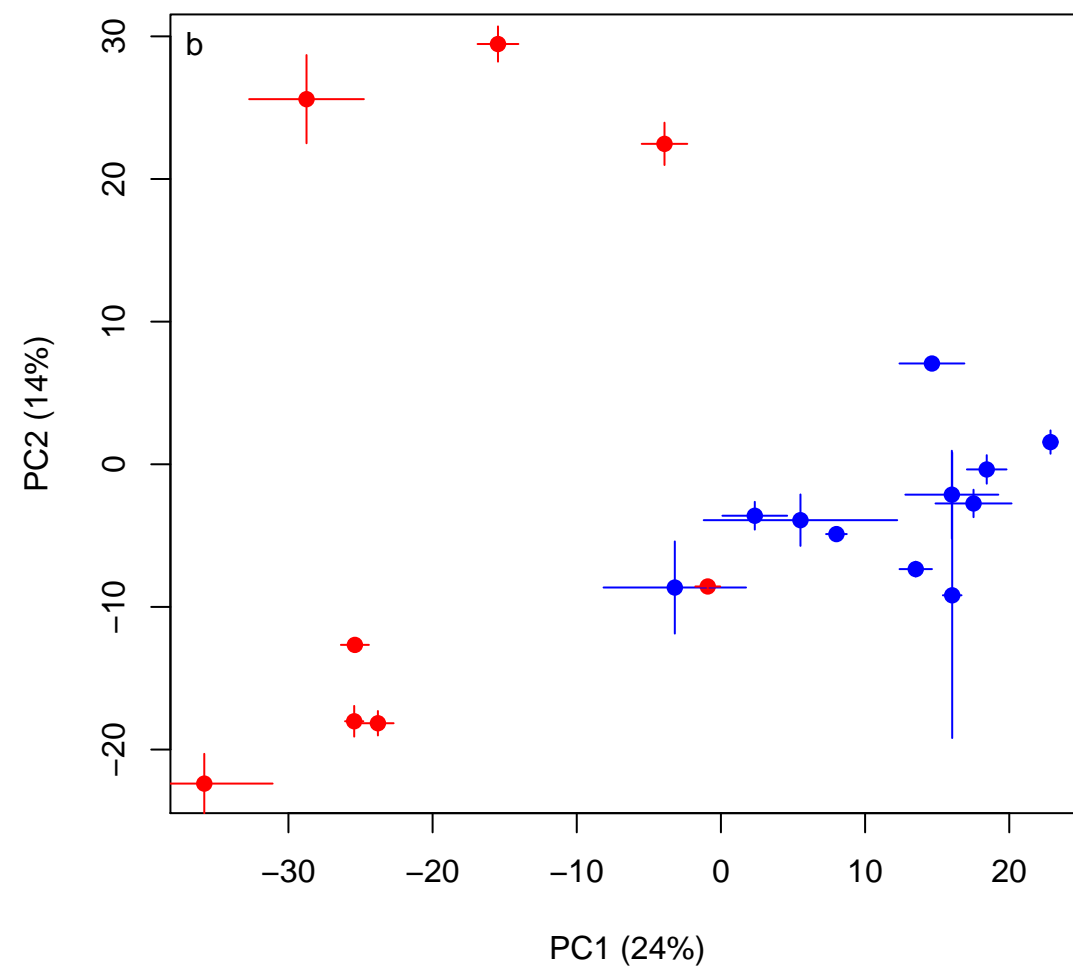

Supplement: Figure S4 — Scatterplots of Bray–Curtis bacterial TRFs demonstrated significant separation along the (A) PCoA 1 axis for both the 2013 (Pseudo F1 = 11.75, PERMANOVA P = 0.0001) and (B) 2015 (Pseudo F1 = 5.13, PERMANOVA P = 0.0001) koala faecal samples. SE bars are representative of combined technical replicates. [file peerj-07-6534-s005.pdf]

2013

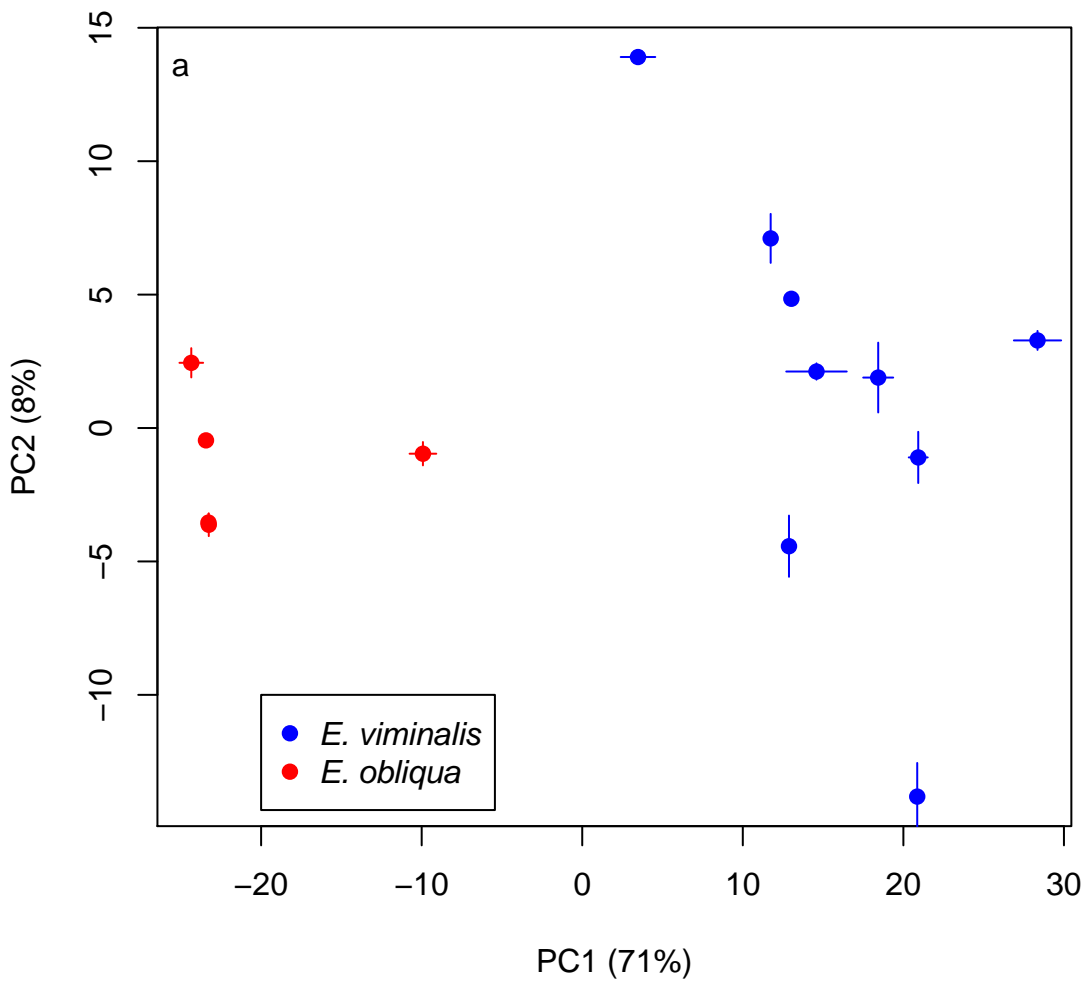

2015

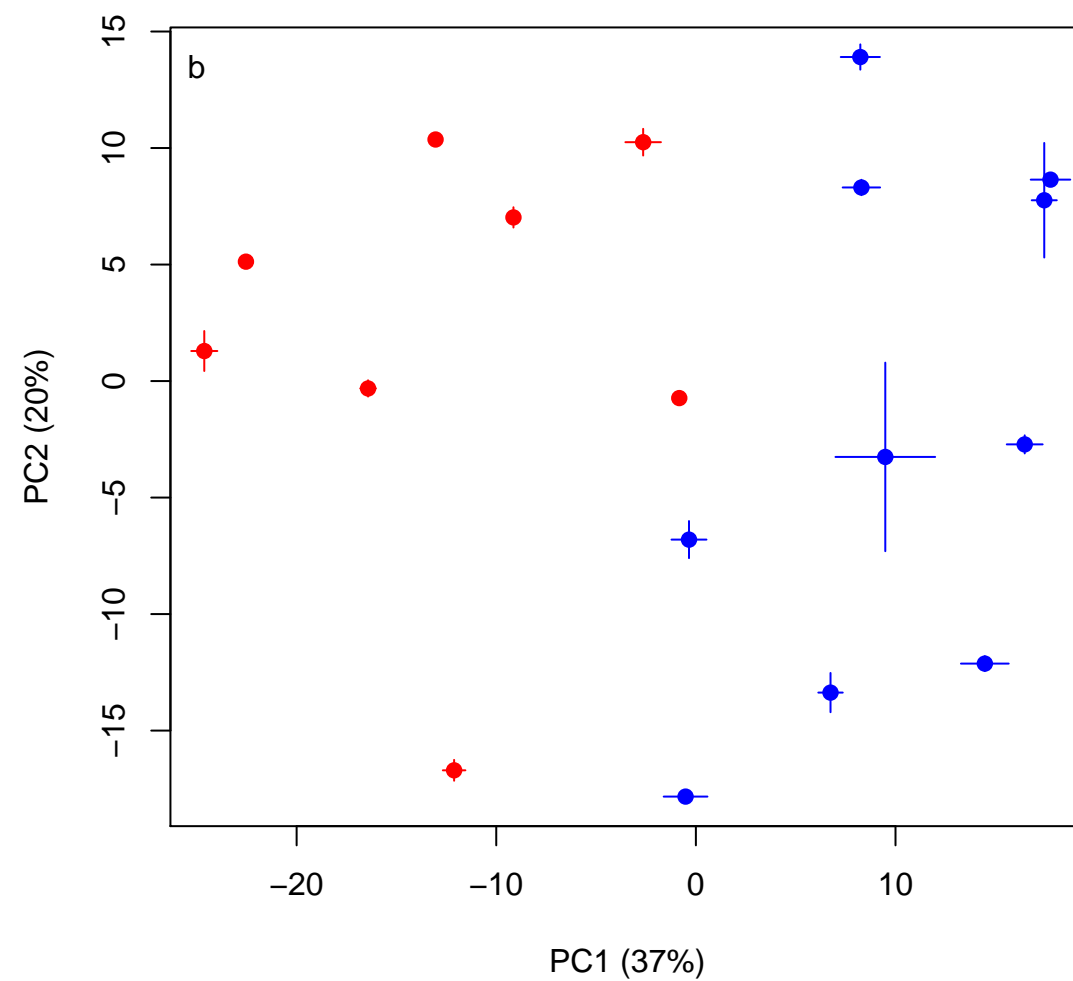

Supplement: Figure S5 — Scatterplots of weighted UniFrac genus-level OTUs from the Cape Otway (A) 2013 and (B) 2015 koala collection years. PERMANOVA analysis of genus-level OTUs revealed a significant separation between koala diets in (A) 2013 (Pseudo F1 = 56.08, PERMANOVA P = 0.001) and (B) 2015 (Pseudo F1 = 17.04, PERMANOVA P = 0.001). [file peerj-07-6534-s006.pdf]

## Archaea

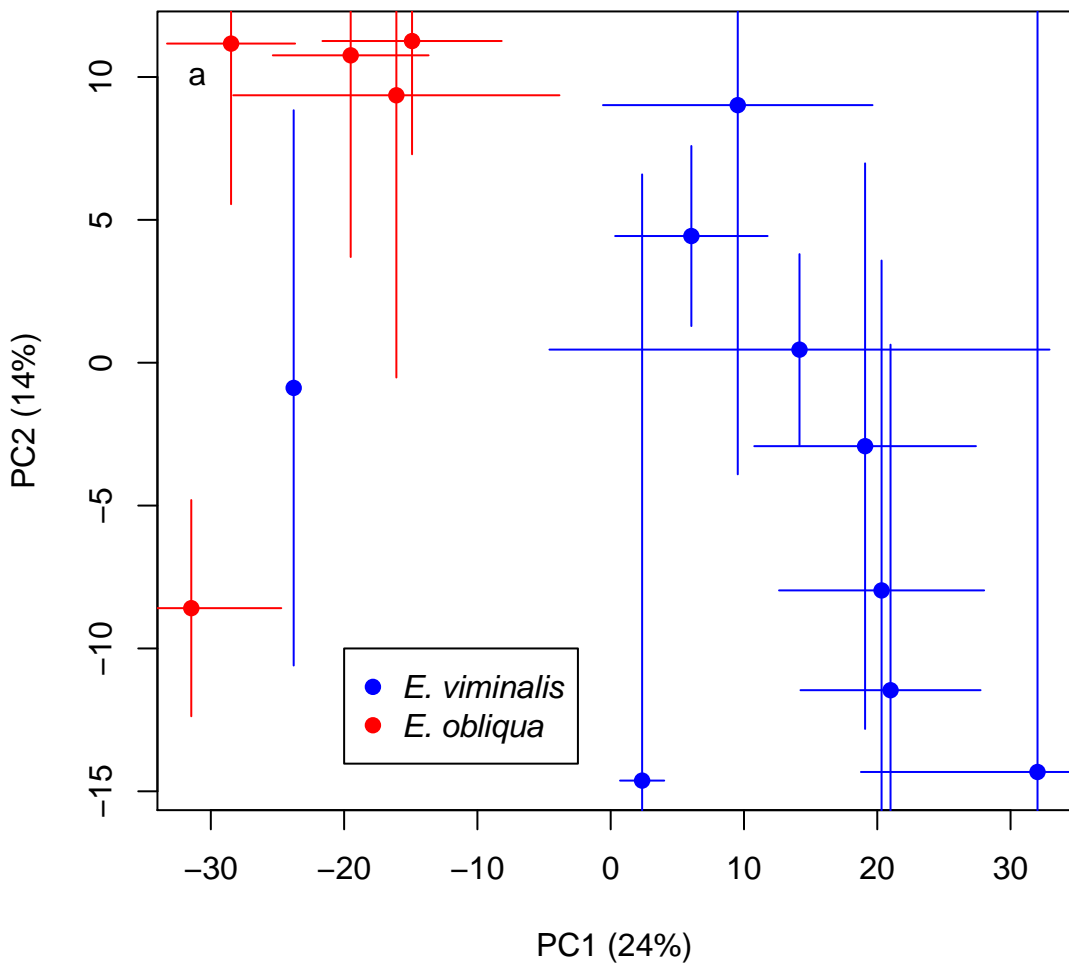

## Fungi

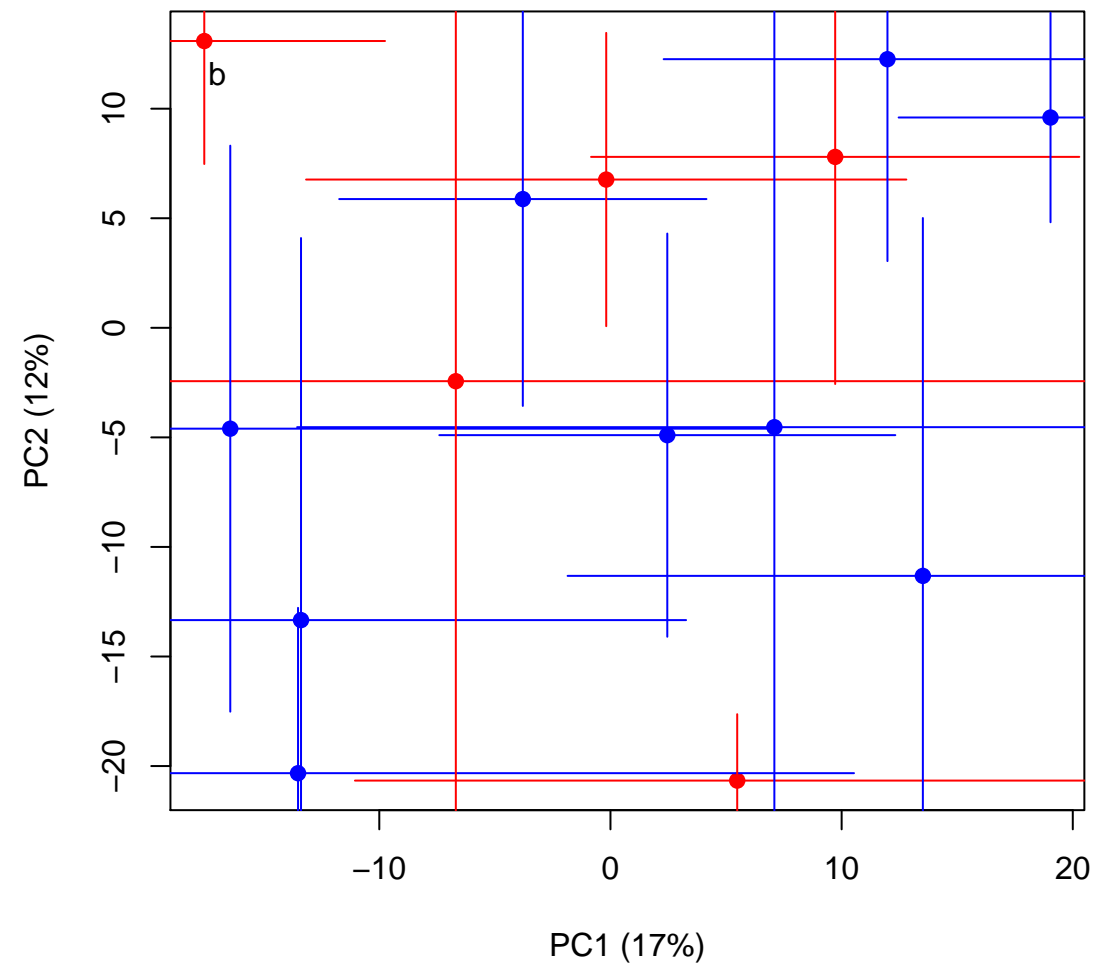

Supplement: Figure S6 — Scatterplots of Bray–Curtis of archaeal 16S and fungal ITS rRNA genes indicated a slight separation along the PCoA axis one for (A) archaea (Pseudo F1 = 4.82, PERMANOVA P = 0.0001. No separation was identified visually by either axis one or two for (B) fungal samples (Pseudo F1 = 2.41, PERMANOVA P = 0.004. SE bars are representative of combined technical replicates. [file peerj-07-6534-s007.pdf]

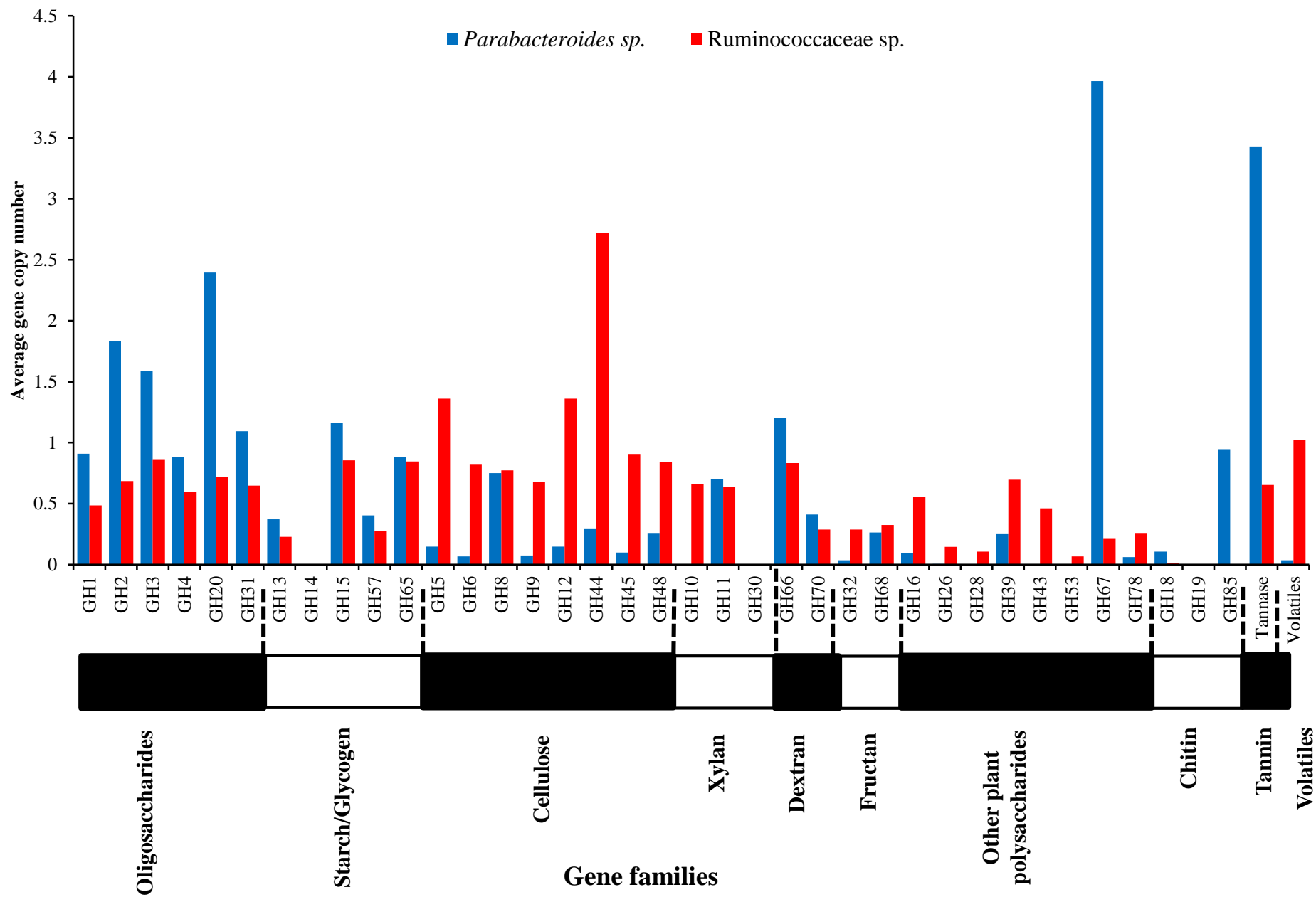

Supplement: Figure S7 — Gene families involved in the metabolism and degradation of plant components i.e., cellulose and the plant secondary metabolite (PSMs) i.e., tannins. [file peerj-07-6534-s008.pdf]

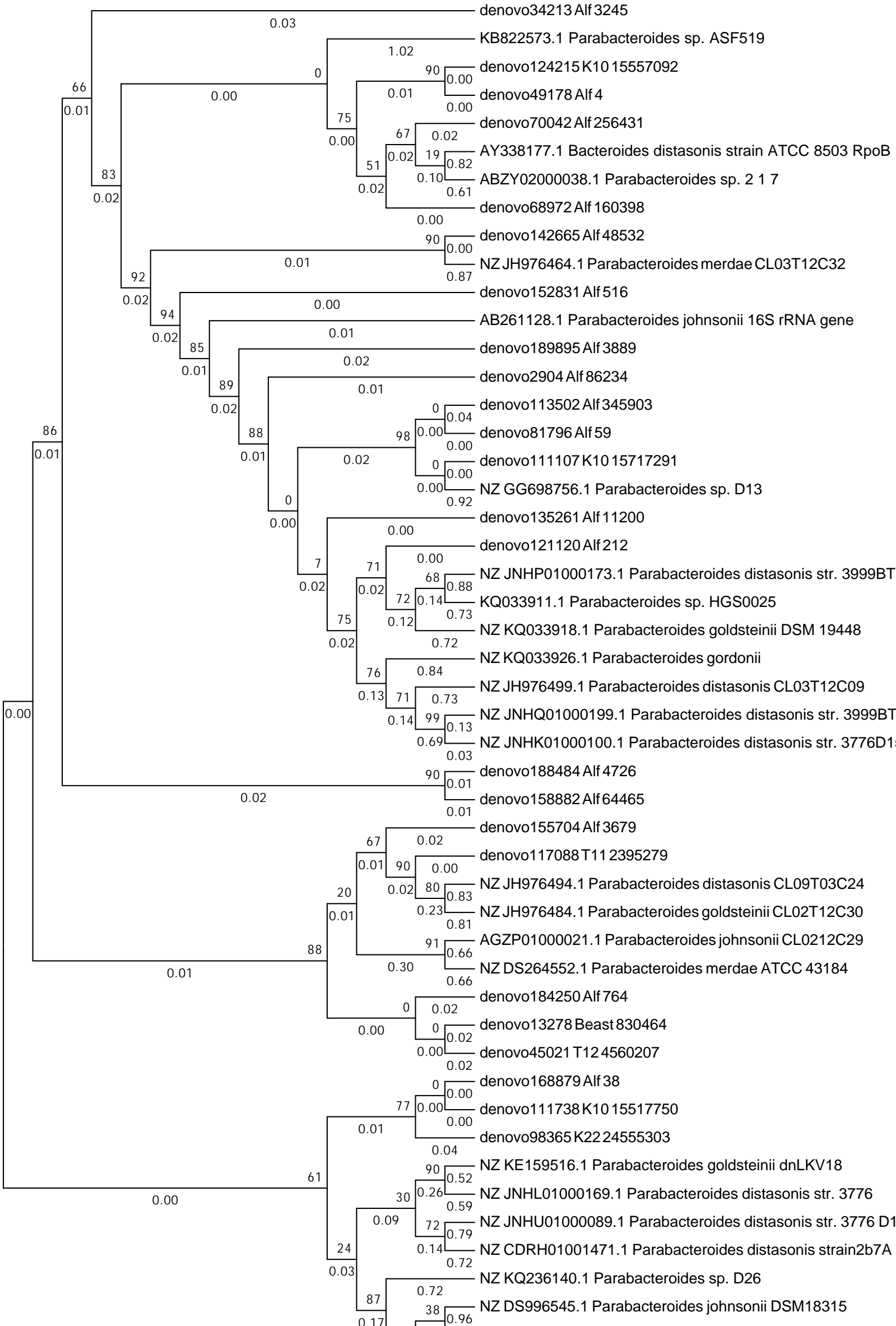

Supplement: Figure S8 — The Parabacteroides phylogenetic tree indicates that the 16S rRNA gene sequences isolated from the koala gut microbiomes are dispersed throughout the Parabacteroides tree. The aLRT statistical test for branches values are indicated on each branch. [file peerj-07-6534-s009.pdf]

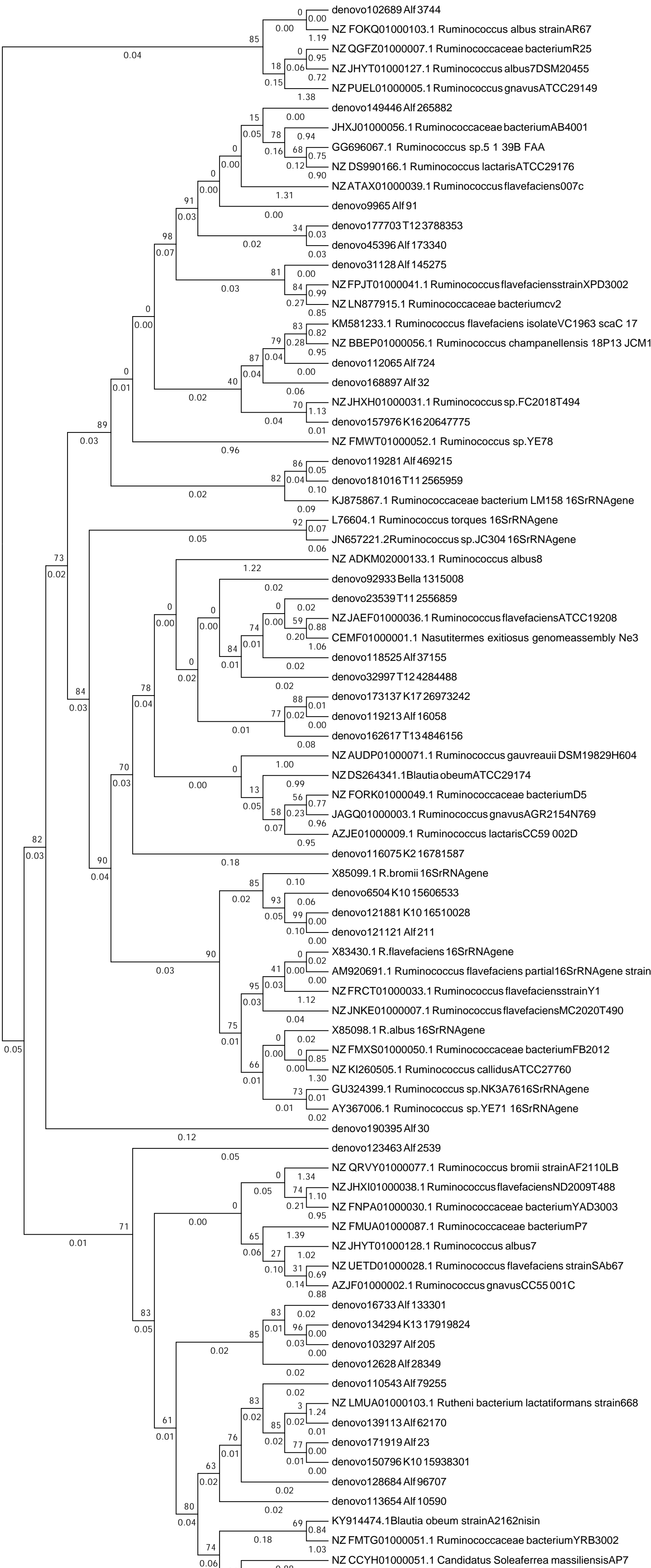

Supplement: Figure S9 — The Ruminococcaceae phylogenetic tree indicates that the 16S rRNA gene sequences isolated from the koala gut microbiomes are dispersed throughout the Ruminococcaceae tree. The aLRT statistical test for branches values are indicated on each branch. [file peerj-07-6534-s010.pdf]
