# Supplementary material for: The Koala (Phascolarctos cinereus) faecal microbiome differs with diet in a wild population
Source: PeerJ. 2019 Apr 1;7:e6534. doi: 10.7717/peerj.6534 (PMC6448554; doi:10.7717/peerj.6534)
Supplement: Tables S1 — Statistics are provided for the combined Cape Otway population sequences processed through QIIME together after split_libraries.py command to produce the seqs.fna file and then after identify_chimeric_seqs.py commands. The sequences were then split into the 2013 and 2015 collections to be analysed separately for OTUs and taxonomic differences and similarities. [file peerj-07-6534-s011.docx]

| **Collection** | **Total sequence number** | **Sequences after chimera slaying** | **mean length ± SE** |
| --- | --- | --- | --- |
| Combined Cape Otway population | 33,102,252 | 31,832,423 | 273 ± 20 |
| 2013 collection | 33,102,252 | 14,582,416 | 253 ± 9 |
| 2015 collection | 33,102,252 | 17,250,007 | 291 ± 7 |
