# Supplementary material for: The Koala (Phascolarctos cinereus) faecal microbiome differs with diet in a wild population
Source: PeerJ. 2019 Apr 1;7:e6534. doi: 10.7717/peerj.6534 (PMC6448554; doi:10.7717/peerj.6534)
Supplement: Table S2A — Alpha diversity indices obtained through QIIME “alpha_diversity.py” command. Diversity indices were then analysed per koala. The National Center for Biotechnology Information, Sequence Read Archive (NCBI SRA) accession number assigned to sequences from each koala are provided. [file peerj-07-6534-s012.docx]

| **Koala** | **Sequence number after chimera removal** | **Shannon** | **Chao 1** | **Diet** | **Collection** | **Accession number** |
| --- | --- | --- | --- | --- | --- | --- |
| Alf | 276,641 ± 34,402 | 4.83 ± 0.03 b b | 6326 ± 331 b b | *E. viminalis* | 2013 | SRX5359014 |
| Beast | 267,891 ± 42,539 | 3.51 ± 0.24 b b | 3670 ± 248 b b | *E. viminalis* | 2013 | SRX5359015 |
| Bella | 208,709 ± 77,592 | 4.05 ± 0.27 b b | 4227 ± 860 b b | *E. viminalis* | 2013 | SRX5359012 |
| Bilko | 288,799 ± 4,328 | 4.35 ± 0.10 b b | 5427 ± 41 b b | *E. viminalis* | 2013 | SRX5359013 |
| T11 | 337,217 ± 11,867 | 4.79 ± 0.07 b b | 6530 ± 505 b b | *E. viminalis* | 2013 | SRX5359018 |
| T12 | 454,493 ± 49,553 | 5.14 ± 0.04 b b | 7258 ± 210 b b | *E. viminalis* | 2013 | SRX5359019 |
| T13 | 367,165 ± 61,708 | 4.10 ± 0.10 b b | 5029 ± 381 b b | *E. viminalis* | 2013 | SRX5359016 |
| T14 | 370,344 ± 34,058 | 4.55 ± 0.15 b b | 6261 ± 633 b b | *E. viminalis* | 2013 | SRX5359017 |
| T15 | 344,484 ± 0 | 4.69 ± 0.00 b b | 5850 ± 0 b b | *E. viminalis* | 2013 | SRX5359010 |
| T17 | 635,440 ± 80,184 | 5.22 ± 0.02a a | 8870 ± 67a a | *E. obliqua* | 2013 | SRX5359011 |
| T18A | 599,340 ± 33,480 | 5.09 ± 0.07a a | 7676 ± 499a a | *E. obliqua* | 2013 | SRX5358990 |
| T18C | 425,727 ± 10,209 | 4.99 ± 0.04a a | 6736 ± 230a a | *E. obliqua* | 2013 | SRX5358991 |
| T19 | 492,525 ± 52,166 | 5.60 ± 0.04a a | 8839 ± 234a a | *E. obliqua* | 2013 | SRX5358992 |
| T20 | 492,476 ± 63,934 | 5.61 ± 0.08a a | 8223 ± 407a a | *E. obliqua* | 2013 | SRX5358993 |
| K6 | 299,307 ± 23,786 | 4.98 ± 0.09 b b | 10621 ± 745 b b | *E. viminalis* | 2015 | SRX5358994 |
| K9 | 374,128 ± 47,782 | 4.94 ± 0.28 b b | 10731 ± 435 b b | *E. viminalis* | 2015 | SRX5358995 |
| K10 | 425,353 ± 38,027 | 4.77 ± 0.09 b b | 12508 ± 315 b b | *E. viminalis* | 2015 | SRX5358996 |
| K12 | 269,608 ± 8,842 | 4.67 ± 0.09 b b | 10321 ± 1475 b b | *E. viminalis* | 2015 | SRX5358997 |
| K13 | 299,590 ± 54,648 | 5.04 ± 0.04 b b | 11382 ± 58 b b | *E. viminalis* | 2015 | SRX5358988 |
| K14 | 275,681 ± 45,786 | 4.83 ± 0.14 b b | 10163 ± 1012 b b | *E. viminalis* | 2015 | SRX5358989 |
| K15 | 336,052 ± 75,033 | 4.71 ± 0.11 b b | 8692 ± 478 b b | *E. viminalis* | 2015 | SRX5359001 |
| K19 | 311,300 ± 10,2907 | 4.27 ± 0.11 b b | 9191 ± 195 b b | *E. viminalis* | 2015 | SRX5359000 |
| K20 | 288,911 ± 15,690 | 4.81 ± 0.04 b b | 9837 ± 216 b b | *E. viminalis* | 2015 | SRX5359003 |
| K21 | 158,966 ± 2,907 | 4.09 ± 0.10 b b | 9863 ± 1286 b b | *E. viminalis* | 2015 | SRX5359002 |
| K1 | 356,752 ± 23,692 | 5.478 ± 0.07a a | 12360 ± 475a a | *E. obliqua* | 2015 | SRX5359005 |
| K2 | 355,916 ± 15,776 | 5.37 ± 0.07a a | 13209 ± 737a a | *E. obliqua* | 2015 | SRX5359004 |
| K3 | 347,992 ± 60,158 | 5.63 ± 0.07a a | 10640 ± 372a a | *E. obliqua* | 2015 | SRX5359007 |
| K4 | 261,575 ± 21,694 | 5.44 ± 0.06a a | 12341 ± 349a a | *E. obliqua* | 2015 | SRX5359006 |
| K5 | 342,451 ± 30,652 | 5.26 ± 0.06a a | 10589 ± 109a a | *E. obliqua* | 2015 | SRX5359009 |
| K16 | 397,815 ± 13,5054 | 4.83 ± 0.17a a | 10367 ± 738a a | *E. obliqua* | 2015 | SRX5359008 |
| K17 | 382,899 ± 30,992 | 5.24 ± 0.06a a | 12423 ± 598a a | *E. obliqua* | 2015 | SRX5358998 |
| K22 | 310,763 ± 41,972 | 5.13 ± 0.07a a | 11797 ± 2186a a | *E. obliqua* | 2015 | SRX5358999 |
