# Supplementary material for: The Koala (Phascolarctos cinereus) faecal microbiome differs with diet in a wild population
Source: PeerJ. 2019 Apr 1;7:e6534. doi: 10.7717/peerj.6534 (PMC6448554; doi:10.7717/peerj.6534)
Supplement: Table S2B — Alpha diversity indices obtained through QIIME “alpha_diversity.py” command. Diversity indices were then analysed per diet. [file peerj-07-6534-s013.docx]

| **Collection/diet** | **Sequence number after chimera removal** | **Shannon** | **Chao 1** |
| --- | --- | --- | --- |
| *E. viminalis* | 15,627,392 ± 71,648 | 4.60 ± 0.41b | 8099 ± 2652b |
| *E. obliqua* | 16,205,031 ± 11,070 | 5.30 ± 0.25a | 10313 ± 2079a |
| *E. viminalis* 2013 | 6,645,884 ± 72,072 | 4.45 ± 0.50b | 5620 ± 1151c |
| *E. obliqua* 2013 | 7,936,532 ± 86,033 | 5.30 ± 0.28a | 8069 ± 892b |
| *E. viminalis* 2015 | 8,981,508 ± 69,931 | 4.71 ± 0.31b | 10331 ± 1083a |
| *E. obliqua* 2015 | 8,268,499 ± 42,474 | 5.30 ± 0.25a | 11716 ± 1054a |
