# Supplementary material for: The Koala (Phascolarctos cinereus) faecal microbiome differs with diet in a wild population
Source: PeerJ. 2019 Apr 1;7:e6534. doi: 10.7717/peerj.6534 (PMC6448554; doi:10.7717/peerj.6534)
Supplement: Table S2C — Alpha diversity indices obtained through QIIME “alpha_diversity.py” command. Diversity indices were then analysed per collection year. [file peerj-07-6534-s014.docx]

| **Collection** | **Sequence number after chimera removal** | **Shannon** | **Chao 1** |
| --- | --- | --- | --- |
| 2013 collection | 14,582,416 ± 12,601 | 4.75 ± 0.60a | 6494 ± 1594b |
| 2015 collection | 17,250,007 ± 61,347 | 4.97 ± 0.41a | 10946 ± 1257a |
