# Supplementary material for: The Koala (Phascolarctos cinereus) faecal microbiome differs with diet in a wild population
Source: PeerJ. 2019 Apr 1;7:e6534. doi: 10.7717/peerj.6534 (PMC6448554; doi:10.7717/peerj.6534)
Supplement: Table S3 — The top 22 bacteria identified to genus level from Cape Otway koalas in eating E. viminalis and E. obliqua in 2013 and 2015. A relative abundance per eucalypt species and total relative abundance for each population is provided. Data obtained from rarefied and summarized genus level OTU tables generated through QIIME. Mean abundance ± 1 SE. [file peerj-07-6534-s015.docx]

| **Frequency (%) ± SE** | | | | |
| --- | --- | --- | --- | --- |
| **Taxon-genus** | ***E. viminalis*  2013** | ***E. obliqua* 2013** | ***E. viminalis*  2015** | ***E. obliqua*  2015** |
| p_Bacteroidetes;c_Bacteroidia;o_Bacteroidales;f_Porphyromonadaceae;g_*Parabacteroides* | 52 ± 0 a | 5 ± 0c | 22 ± 0b | 19 ± 04b |
| p_Firmicutes;c_Clostridia;o_Clostridiales;f_Ruminococcaceae;g_ | 14 ± 0c | 45 ± 0a | 12 ± 0c | 31 ± 04b |
| p_Bacteroidetes;c_Bacteroidia;o_Bacteroidales;f_Bacteroidaceae;g_*Bacteroides* | 7 ± 02b | 14 ± 0ab | 25 ± 0b | 7 ± 0b |
| p_Firmicutes;c_Clostridia;o_Clostridiales;f_Ruminococcaceae;g_*Ruminococcus* | 13 ± 0a | 2 ± 0b | 9 ± 0a | 3 ± 0b |
| p_Firmicutes;c_Clostridia;o_Clostridiales;f_Lachnospiraceae;g_ | 3 ± 0b | 11 ± 0a | 4 ± 0b | 8 ± 0a |
| p_Firmicutes;c_Clostridia;o_Clostridiales;f_;g_ | 1 ± 0b | 7 ± 0ab | 2 ± 0b | 11 ± 0a |
| p_Cyanobacteria;c_4C0d-2;o_YS2;f_;g_ | 1 ± 0b | 6 ± 0ab | 16 ± 0a | 8 ± 0ab |
| p_Verrucomicrobia;c_Verrucomicrobiae;o_Verrucomicrobiales;f_Verrucomicrobiaceae;g_*Akkermansia* | 2 ± 0a | 0.00004 ± 0.00002a | 1 ± 0a | 1 ± 0a |
| p_Bacteroidetes;c_Bacteroidia;o_Bacteroidales;f_Rikenellaceae;g_ | 1 ± 0ab | 3 ± 0a | 1 ± 0ab | 1 ± 0b |
| Unassigned;Other;Other;Other;Other;Other | 1 ± 0b | 1 ± 0a | 0.003 ± 0.001c | 0.003 ± 0.001c |
| p_Firmicutes;c_Erysipelotrichi;o_Erysipelotrichales;f_Erysipelotrichaceae;g_*Coprobacillus* | 1 ± 0ab | 2 ± 0a | 0.001 ± 0.0002b | 0.002 ± 0.0003b |
| p_Firmicutes;c_Clostridia;o_Clostridiales;f_Ruminococcaceae;g_*Oscillospira* | 0.003 ± 0.001ab | 1 ± 01a | 1 ± 0b | 0.001 ± 0.0003b |
| p_Proteobacteria;c_Gammaproteobacteria;o_Enterobacteriales;f_Enterobacteriaceae;g_ | 0.003 ± 0.002a | 0.003 ±0.001a | 0.0003 ± 0.0001a | 0.001 ± 0.0004a |
| p_Firmicutes;c_Clostridia;o_Clostridiales;f_Clostridiaceae;g_*Clostridium* | 1 ± 0a | 0.0004 ± 0.0003b | 1 ± 02a | 0.003 ± 0.001b |
| p_Firmicutes;c_Clostridia;o_Clostridiales;Other;Other | 0.001 ± 0.0001b | 0.004 ± 0.0004a | 0.002 ± 0.0002b | 0.004 ± 0.001a |
| p_Firmicutes;c_Clostridia;o_Clostridiales;f_Veillonellaceae;g_*Acidaminococcus* | 0.002 ± 0.0005b | 0.003 ± 0.001b | 02 ± 0a | 1 ± 0ab |
| p_Proteobacteria;c_Betaproteobacteria;o_Burkholderiales;f_Alcaligenaceae;g_*Sutterella* | 0.002 ± 0.001a | 0.003 ± 0.001a | 0.002 ± 0.001a | 0.002 ± 0.0004a |
| p_Proteobacteria;c_Deltaproteobacteria;o_Desulfovibrionales;f_Desulfovibrionaceae;g_ | 0.002 ± 0.0004a | 0.002 ± 0.0003a | 0.002 ± 0.0003a | 0.001 ± 0.0002a |
| p_Firmicutes;c_Clostridia;o_Clostridiales;f_Lachnospiraceae;Other | 0.001 ± 0.0001c | 0.003 ± 0.0003ab | 0.002 ± 0.0002bc | 0.004 ± 0.001c |
| p_Synergistetes;c_Synergistia;o_Synergistales;f_Synergistaceae;Other | 0.003 ± 0.001b | 0.0003 ± 0.0001b | 2 ± 0a | 1 ± 0b |
| p_Firmicutes;c_Clostridia;o_Clostridiales;f_Lachnospiraceae;g_*Butyrivibrio* | 0.0006 ± 0.0001a | 0.001 ± 0.0004a | 0.002 ± 0.0004a | 0.002 ± 0.0003a |
