# Supplementary material for: The Koala (Phascolarctos cinereus) faecal microbiome differs with diet in a wild population
Source: PeerJ. 2019 Apr 1;7:e6534. doi: 10.7717/peerj.6534 (PMC6448554; doi:10.7717/peerj.6534)
Supplement: Table S4 — Identification of the top 24 bacterial families identified by “summarise_taxa.py” OTUs command in QIIME for Cape Otway koalas in 2013 and 2015. Relative abundance is provided by diet, data obtained from rarefied and summarized family level OTU tables generated through QIIME. Mean abundance ±1 SE. [file peerj-07-6534-s016.docx]

| **Frequency (%) ± SE** | | | | |
| --- | --- | --- | --- | --- |
| **Taxon-family** | ***E. viminalis* 2013** | ***E. obliqua*  2013** | ***E. viminalis* 2015** | ***E. obliqua* 2015** |
| p_Firmicutes;c_Clostridia;o_Clostridiales;f_Ruminococcaceae | 27 ± 0b | 48 ± 0a | 21 ± 0c | 34 ± 0ab |
| p_Bacteroidetes;c_Bacteroidia;o_Bacteroidales;f_Porphyromonadaceae | 53 ± 0a | 5 ± 0c | 24 ± 0b | 19 ±0b |
| p_Bacteroidetes;c_Bacteroidia;o_Bacteroidales;f_Bacteroidaceae | 7 ± 0b | 14 ± 0ab | 22 ± 0a | 7 ± 0b |
| p_Firmicutes;c_Clostridia;o_Clostridiales;f_Lachnospiraceae | 4± 0b | 12 ± 0a | 5 ± 0b | 12 ± 0a |
| p_Firmicutes;c_Clostridia;o_Clostridiales;f_ | 1 ± 0b | 7 ± 0ab | 2 ± 0b | 11 ± 0a |
| p_Cyanobacteria;c_4C0d-2;o_YS2;f_ | 1 ± 0b | 6 ± 0 ab | 15 ± 0a | 8 ± 0ab |
| p_Verrucomicrobia;c_Verrucomicrobiae;o_Verrucomicrobiales;f_Verrucomicrobiaceae | 2 ± 0a | 0.00004 ± 0.00002a | 1 ± 0a | 1 ± 0a |
| p_Bacteroidetes;c_Bacteroidia;o_Bacteroidales;f_Rikenellaceae | 1 ± 0ab | 3 ± 0a | 1 ± 0ab | 1 ± 0b |
| Unassigned;Other;Other;Other;Other | 1 ± 0b | 1 ± 0a | 0.003 ±0.001c | 0.003 ± 0.001c |
| p_Firmicutes;c_Clostridia;o_Erysipelotrichales;f_Erysipelotrichaceae | 1 ± 0ab | 2 ± 0a | 0.002 ± 0.001b | 0.002 ± 0.0003b |
| p_Proteobacteria;c_Deltaproteobacteria;o_Desulfovibrionales;f_Desulfovibrionaceae | 1 ± 01a | 0.003 ± 0.0002a | 0.005 ± 0.001a | 0.003 ± 0.0003a |
| p_Firmicutes;c_Clostridia;o_Clostridiales;f_Veillonellaceae | 0.004 ± 0.001a | 0.003 ± 0.001a | 3 ± 0a | 3 ± 0a |
| p_Proteobacteria;c_Gammaproteobacteria;o_Enterobacteriales;f_Enterobacteriaceae | 0.003 ± 0.002a | 0.003 ± 0.001a | 0.0004 ± 0.0001a | 0.001 ± 0.0004a |
| p_Firmicutes;c_Clostridiao_Clostridiales;f_Clostridiaceae | 0.005 ± 0.001b | 0.0004 ± 0.0003b | 1 ± 0a | 0.003 ± 0.001b |
| p_Firmicutes;c_Clostridia;o_Clostridiales;Other | 0.001 ± 0.0001b | 0.004 ± 0.0004a | 0.002 ± 0.0002b | 0.004 ± 0.0001a |
| p_Proteobacteria;c_Betaproteobacteria;o_Burkholderiales;f_Alcaligenaceae | 0.002 ± 0.001a | 0.003 ± 0.001a | 0.002 ± 0.001a | 0.002 ± 0.0004a |
| p_Synergistetes;c_Synergistia;o_Synergistales;f_Synergistaceae | 0.003 ± 0.001b | 0.0004 ± 0.0001b | 2 ± 03a | 0 ± 0b |
| p_Planctomycetes;c_vadinHA49;o_PeHg47;f_ | 0.001 ± 0.0001ab | 0.003 ± 0.001 a | 0.001 ± 0.001ab | 1 ± 0b |
| p_Fusobacteria;c_Fusobacteriia;o_Fusobacteriales;f_Fusobacteriaceae | 0.001 ± 0.001a | 0.002 ± 0.001a | 0.004 ± 0.001a | 0.0001 ± 0.0003a |
